# Who Pays, Who Gains? ACA Medicaid Expansions and Changes in Net Income and Tax Liability by Socioeconomic Status

**DOI:** 10.1111/1475-6773.70147

**Published:** 2026-07-08

**Authors:** Matthew Hui Liu, Rebecca Anna Schut

**Affiliations:** ^1^ School of Medicine, Case Western Reserve University Cleveland Ohio USA; ^2^ Department of Agricultural Economics, Sociology, and Education The Pennsylvania State University University Park Pennsylvania USA

## Abstract

**Objective:**

To evaluate the effects of Affordable Care Act (ACA) Medicaid expansions on changes to net (after‐tax) income and federal tax liability across socioeconomic status (SES).

**Study Setting and Design:**

Event study and difference‐in‐difference analyses assessed the effects of state‐level ACA Medicaid expansions post‐2020 on net income and federal tax liability.

**Data Sources and Analytic Sample:**

Analyses were performed on nationally‐representative survey data from the 2010 to 2023 Integrated Public Use Microdata Series Current Population Survey (IPUMS CPS) Annual Social and Economic Supplement (ASEC). We restricted the sample to individuals aged 18–65 with known information on sociodemographic characteristics.

**Principal Findings:**

Medicaid expansions contributed to overall net income growth (2.10%, 95% CI 1.31–2.89). By socioeconomic status, postexpansion net incomes were higher among individuals in the lowest income quintile (3.56%, 1.47–5.65), among those employed in low (4.90%, 2.74–7.07), low‐mid (2.05%, 0.57–3.54), and mid‐high (1.97%, 0.61–3.32) skill occupations, and among those with less than a high school degree (3.51%, 0.50–6.51), some college (3.39%, 1.47–5.32), or a bachelor's degree (1.94%, 0.32–3.56). Results further show an increase in overall federal tax liability after deductions (3.84%, 2.43–5.25), namely among individuals in the second (5.20%, 0.72–9.68) and third (4.58%, 2.31–6.86) income quintiles, among those in low (6.99%, 2.78–11.20), low‐mid (6.82%, 4.05–9.60), and mid‐high (2.71%, 0.42–5.01) skill occupations, and among those with a high school degree or GED (4.79%, 1.97–7.60) or a master's degree (7.99%, 3.70–12.28).

**Conclusions:**

Findings suggest ACA Medicaid expansions induced downstream net income gains while increasing overall federal tax revenue. Results further suggest that the increased costs of Medicaid expansion are not largely borne by higher SES groups. Broadly, Medicaid expansions may provide substantial economic benefits to the entire U.S. population.

## Callout Box

- What is known on this topic
  ○ Medicaid expansions under the Affordable Care Act (ACA) reduced the proportion of the U.S. population without health insurance coverage and has been shown to improve health and healthcare utilization, and reduce mortality, particularly among low‐income Americans.
  ○ Medicaid expansion has also been shown to confer substantial economic benefits to newly‐eligible Americans as well as certain healthcare workers.
  ○ Investigation into the economic consequences of expansion across SES groups remains limited. Whether and for whom expansion has increased federal tax liability is currently unknown.
- What this study found
  ○ ACA Medicaid expansions induced net (after‐tax) income growth. Results suggest a significant increase in overall federal tax liability, but this did not translate to significant reductions in net income among higher SES groups.
  ○ Findings suggest that Medicaid expansions provide financial benefits extending beyond low SES groups, while the resultant costs of expansion are borne largely by those most likely to benefit from the program.
  ○ Expansions to the social safety net, including further expansions to healthcare insurance, may provide substantial economic benefits to the entire U.S. population.

## 1 Introduction

Medicaid expansions under the Affordable Care Act (ACA) have had substantial effects on the well‐being of the U.S. population. Most directly, expansions have reduced the proportion of the U.S. population without health insurance coverage [1], and have improved outcomes in health, healthcare utilization, and mortality, particularly among low‐income Americans [2, 3, 4, 5, 6, 7, 8, 9, 10, 11, 12, 13, 14, 15, 16]. Recent scholarship has further explored whether and to what degree Medicaid expansion shapes population outcomes outside of health, investigating its effects on crime and arrest rates [17, 18], homelessness [19], and educational outcomes [20, 21]. Additionally, researchers have aimed to understand the *political economy* of Medicaid expansion through exploring its role in shaping a range of labor market outcomes, particularly among new Medicaid enrollees [16, 22]. Notably, newly‐eligible Americans experience wage increases as a result of expansion, suggesting that improved access to health care resulting from Medicaid coverage confers substantial economic benefits to lower socioeconomic status (SES) individuals [23]. Other research suggests industry‐specific income gains, identifying growth in earnings among the highest‐earning healthcare workers in expansion states (e.g., physicians, executives, and some nurses) [24].

Still, investigation into whether ACA Medicaid expansions hold broader economic consequences for the population and across SES remains limited. Research has documented important “spillover effects” occurring as a result of other social safety net policies, including minimum wage increases [25, 26] and the Earned Income Tax Credit program [27, 28]. Such findings suggest that Medicaid expansion may also impact individuals beyond those who are newly‐eligible for Medicaid. Namely, we argue Medicaid expansion may generate important changes to state‐ and local‐level labor markets that shape working conditions and improve earnings as a result [15]. Because Medicaid expansion decouples access to health insurance and health services from employment, workers—particularly those who receive Medicaid—may be able to more freely move between jobs, weakening job lock and placing pressure on employers to raise wages across the board [29]. Expansion may also stimulate local‐level economic development, bolstering local health care economies and other related economic sectors, creating more employment options and potentially generating higher wages for workers, especially in rural areas of the U.S. [24].

Moreover, because expansion has been shown to improve health [2, 3, 4, 5, 6, 7, 8, 9, 10, 11, 12, 13, 14, 15, 16], workers who possess Medicaid may experience reductions in absenteeism, unemployment, and economic inactivity, resulting in lower turnover and higher levels of productivity [30], which can drive up wages. In terms of health care costs specifically, because expansion prevents medical cost shifting [31], insurance premiums may decrease, resulting in increases to real wages for even workers holding private insurance. As a result, Medicaid expansion may improve earnings not only for those who are direct beneficiaries, but also for non‐Medicaid enrolled individuals.

Furthermore, we argue expansion may induce important changes in tax liability. Indeed, whether and for whom expansion has increased federal tax liability—that is, the amount one owes to the federal government in taxes—remains understudied to our knowledge. We hypothesize that the same labor market spillovers that result in higher wages may also affect nonbeneficiaries, raising wages, which may expand and bolster states' tax bases and result in higher tax liabilities. Moreover, examining how Medicaid expansion affects federal tax liability is critical, given longstanding political debates regarding *who* should ultimately be responsible for financing social safety net programs [32, 33]. Taken together, we argue Medicaid expansion may therefore serve as not only an important *health* policy, but an *economic* one as well.

This study investigated the economic effects of Medicaid expansion on the U.S. population. We explored both the overall effects of expansion, as well as the effects across SES groups, focusing on two economic outcomes specifically—net income (i.e., after‐tax or “take home” income) and federal tax liability after tax credit deductions. Our analyses further investigated whether and for which SES groups Medicaid expansion has conferred economic benefits, and on whom the responsibility for funding an expanded social safety net falls. Findings may hold critical implications for our understanding of the social and economic externalities associated with safety net program expansions. Whether Medicaid expansion has a “spillover effect” on changes in the income distribution and tax base of the U.S. more broadly may have far‐reaching impacts for not only the overall economy, but also for the financial well‐being of the U.S. healthcare system [34]. Improved economic outcomes for the population may also promote population health and health equity [35].

## 2 Methods

### 2.1 Data and Sample

Our analyses drew on publicly‐available microdata from the 2010 to 2023 Integrated Public Use Microdata Series Current Population Survey (CPS) Annual Social and Economic Supplement (ASEC) [36]. We restricted the sample to individuals aged 18–65 at the time of survey with known respondent‐reported race/ethnicity, sex, nativity (i.e., U.S. or foreign born), and marital status. Our final analytical sample included 1,242,634 individuals across the 50 states and the District of Columbia (D.C.). We identified states' Medicaid expansion status via information available through the Kaiser Family Foundation [1].

### 2.2 Exposure

**FIGURE 1:**
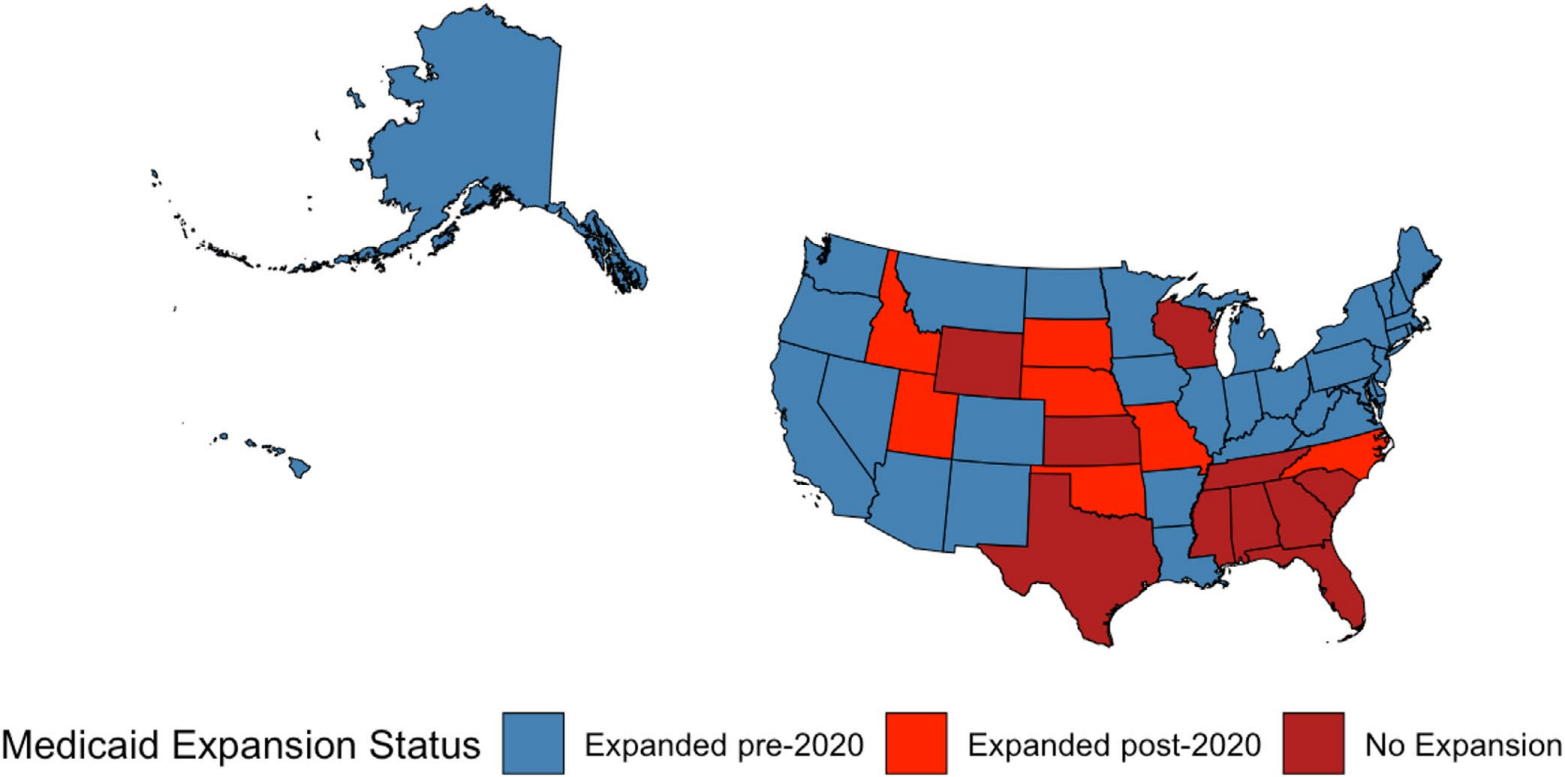
Medicaid expansion status by state. Respondents living in states that never expanded Medicaid at any point were excluded from event study analysis. In DiD analyses, states that expanded Medicaid prior to 2020 were counted as expansion states, whereas states that expanded after 2020 or never expanded were counted as nonexpansion states. Data on expansion year come from the KFF.

Our key exposure was whether respondents resided in a state that expanded Medicaid by the year 2020. Figure 1 shows Medicaid expansion across states before and after 2020. Thirty‐three states and D.C. had expanded Medicaid prior to 2020. Seventeen states either expanded after 2020 or have yet to expand and were therefore considered nonexpansion states for the purpose of this analysis. 2020 was selected to facilitate a long‐term analysis of the economic consequences of Medicaid expansion post‐ACA implementation, but before the onset of the COVID‐19 pandemic. As most states that expanded Medicaid did so on January 1st of a given year [1], individuals residing in states that expanded Medicaid in 2019 would have been exposed to expansion for at least 1 year prior to the beginning of the COVID‐19 pandemic in March 2020.

### 2.3 Outcomes

We considered the effects of ACA Medicaid expansions on two economic outcomes: net (after‐tax) income and federal income tax liability after tax credit deductions. Net income was calculated by subtracting federal tax liability from total earned income. Total earned income was derived directly from the CPS variable “INCTOT” which captures individuals' pretax wages and salary income received from all sources in the calendar year prior to being surveyed. Income is recorded in the CPS data as reported by respondents. While analyses presented in the main text focus on net income after taxes, supplementary analyses (available in Figures S1 and S2) present findings from analyses where total pretax income was used as they key outcome.

Federal income tax liability after credit deductions was derived from the “FEDTAXAC” CPS variable, which indicates the amount of federal tax owed by respondents after tax credits are deducted. These credits include The Additional Child Tax Credit and The Earned Income Tax Credit. Amounts are reported in dollars for the survey year. In 2021 and 2022, the economic impact payments were imputed and included, while the expanded 2021 child credits were imputed for the 2022 CPS ASEC sample [37]. We investigated federal—as opposed to state—tax liability for two reasons. First, only information on federal (and not state) tax liability is collected by the CPS. Second, although Medicaid programs receive a significant proportion of their funding from state taxes, federal funding is also critical. Specifically, the base federal match (FMAP), is the proportion of state spending matched by the federal government for health services. While the base varies by state, by law it cannot exceed 83% or fall below 50% [13].

Both outcomes were inflation adjusted to 2023 dollars using the Consumer Price Index from the Bureau of Labor Statistics. Our analyses were population weighted using the CPS ASEC weights.

We examined changes in net income and federal tax liability overall (for all respondents) and across SES groups. We captured respondents' SES using three separate measures. The first measure grouped respondents into income quintiles; the second captured respondents' highest level of educational attainment using an eight‐category variable (less than high school, high school or GED, some college, associate's degree, bachelor's degree, master's degree, professional degree, and doctorate degree); and the final measure grouped respondents by occupational skill level. Respondents' occupational skill was ascertained using the coding schema developed by the Occupational Information Network (O*NET) Job Skills data [38]. The O*NET data assigns each occupation represented in the CPS a score from 1 to 5, with “1” denoting low skill occupations (e.g., landscaping, housekeeping workers), “2” denoting low‐mid skill occupations (e.g., orderlies, customer service representatives), “3” denoting mid skill occupations (e.g., medical assistants, court reporters), “4” denoting mid‐high skill occupations (e.g., sales managers, graphic designers), and “5” denoting high skill occupations (e.g., physicians, lawyers). Notably, only respondents who reported a valid occupation in the CPS (i.e., employed respondents) were able to be assigned a job zone (64%). Those without a job zone were excluded from our analysis of job zone groups.

Our analysis proceeded in two steps. To observe changes in the outcomes based on years since Medicaid expansion, we performed event study analysis. Years following expansion (designated as year 0) were denoted by the number of years after expansion (1 to 9) or prior to expansion (−4 to −1). Respondents living in states that had not expanded Medicaid or those who were surveyed 5 or more years prior to expansion were excluded from this analysis.

We then used difference‐in‐differences (DiD) models to estimate the effects of Medicaid expansion on net income and federal tax liability after tax credit deductions for all respondents across income quintiles, educational attainment groups, and occupational skill groups. In doing so, we followed the approach of de Chaisemartin and D'Haultfoeuille, which accounts for bias that can arise in standard two‐way fixed‐effect models as a result of staggered policy implementation and heterogeneous treatment effects [39, 40, 41]. DiD models adjusted for several potential confounders including sex (male, female), self‐reported race/ethnicity (white, black, Hispanic, Asian), nativity (U.S. born, foreign born), marital status (single, married with spouse present, married with spouse absent, separated, divorced, and widowed). We include these sociodemographic characteristics given their recognized importance as key stratifying features associated with access to resources—including health insurance/services, wages, education, and occupations—within the U.S. We additionally accounted for occupational skill and educational attainment when these factors were not the main SES measure in the analysis (i.e., in models where income quintile was the key independent variable).

## 3 Results

Table 1 presents weighted descriptive characteristics of the study sample. Individuals living in states that did not expand Medicaid were more likely to live in the U.S. South (73% vs. 17.5%), whereas respondents living in states that did expand Medicaid were most likely to reside in states in the U.S. Northeast (25.6% vs. 0%) and West (34.3% vs. 5.0%). Median age for expansion states and nonexpansion states were the same (45). Respondents in expansion states were more likely to be women (47.8% vs. 47.5%), white (63.0% vs. 62.5%), immigrant (29.5% vs. 22.7%), or never married (29.7% vs. 26.3%). Expansion states had a higher percentage of Medicaid recipients (16.7% vs. 7.8%). On average, respondents in expansion states had higher mean total (pretax) income ($71,243 vs. $63,590), net income ($62,444 vs. $58,580), and greater postdeduction tax liabilities ($7428 vs. $5657).

**TABLE 1:** Weighted descriptive characteristics of respondents in the 2010–2023 IPUMS CPS.

	Med expansion states	Nonexpansion states	All states
Median age	45	45	45
Women (%)	47.8	47.5	47.7
Race/ethnicity (%)			
White	63.0	62.5	62.8
Black	9.8	14.0	11.1
Asian	8.9	3.9	7.2
Hispanic	18.3	19.6	18.7
Nativity (%)			
U.S. born	70.5	77.3	72.8
Immigrant	29.5	22.7	27.2
Marital status (%)			
Married, spouse present	55.2	57.1	55.8
Married, spouse absent	1.5	1.47	1.47
Separated	2.0	2.32	2.10
Divorced	9.9	10.9	10.2
Widowed	1.81	1.96	1.86
Never married/single	29.7	26.3	28.6
Census region (%)			
Northeast	25.6	0.0	18.4
Midwest	22.6	21.5	22.6
South	17.5	73.5	33.6
West	34.3	5.0	25.4
Educational attainment (%)			
Less than high school	7.9	8.9	8.3
High school or GED	27.5	11.6	28.0
Some college	17.4	18.5	17.7
Associate's Degree	10.1	11.6	10.6
Bachelor's Degree	23.0	21.0	22.4
Master's Degree	10.1	7.9	9.4
Professional Degree	1.8	1.3	1.6
Doctorate Degree	2.1	1.7	2.0
Job zone (Occupational skill) (%)			
1 (low skill)	12.7	13.1	12.9
2 (low‐mid skill)	29.5	31.4	30.1
3 (mid skill)	7.9	8.6	8.14
4 (mid‐high skill)	33.6	31.5	32.9
5 (high skill)	16.2	15.4	16.0
Income quintile (%)			
Quintile 1	20.1	19.9	20.0
Quintile 2	18.8	21.2	20.0
Quintile 3	18.7	21.3	20.0
Quintile 4	20.3	19.6	20.0
Quintile 5	22.0	18.0	20.0
Mean total earnings (2023 $)	71,243	63,590	68,713
SE	101.0	129.0	79.8
Mean federal tax liability (2023 $)	7428	5657	6843
SE	26.9	34.1	21.3
Net earnings (2023 $)	62,444	56,580	60,503
SE	82.2	106.0	65.3
*N*	831,282	411,352	1,242,634

**FIGURE 2:**
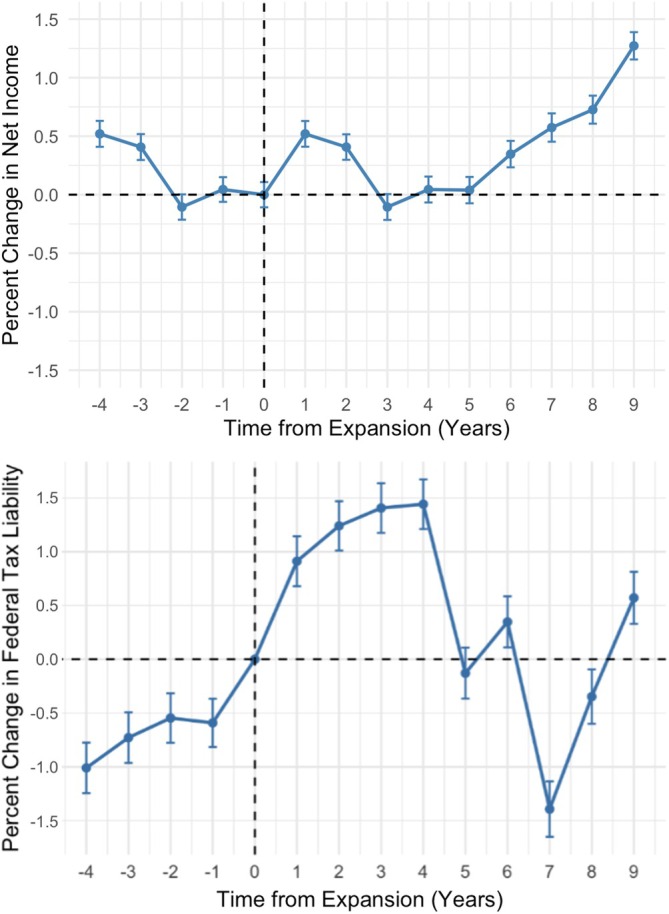
Event study analyses of change in net income and federal tax liability, pre‐ and post‐Medicaid expansion. The top figure shows findings from event study analysis showing percent change in net income, calculated by subtracting total personal income by federal tax liability after tax credit deductions, relative to expansion year for all states that expanded Medicaid. The bottom figure reports findings from event study analysis showing percent change in federal tax liability after tax credit deductions relative to expansion year for all states that expanded Medicaid. The *y*‐axis plots percent difference in income or federal tax liability from the year of expansion for each event time. The *x*‐axis represents the number of years relative to the timing of state Medicaid expansions. The 95% CIs are based on standard errors. The vertical black dotted line represents the year of Medicaid expansion. The horizonal black dotted line represents the income or federal tax liability the year of Medicaid expansion.

Figure 2 presents findings from event study analysis. Event studies performed separately for each SES measure (income quintile, occupation skill, and education level) are available in Figures S3–S5.

Figure 2 Panel 1 shows an increase in net income immediately after expansion that reverts in year 3 but continues to rise from year 6 onward. Figure 2 Panel 2 additionally shows that mean federal tax liability after deductions increased immediately following Medicaid expansion before dipping dramatically 5 years postexpansion, such that change in mean tax liability in years 5 and 7 were negative. As most states that expanded Medicaid did so in 2014, the drop in tax liability in year 5 likely reflects the 2018 Tax Cuts and Jobs Act, which reduced statutory tax rates at almost all levels of taxable income [42]. The drop in liability observed in year 7 likely corresponds to the numerous tax credits implemented in response to the COVID‐19 pandemic (i.e., 2020 and 2021) and the associated recession [43, 44, 45, 46, 47, 48].

**FIGURE 3:**
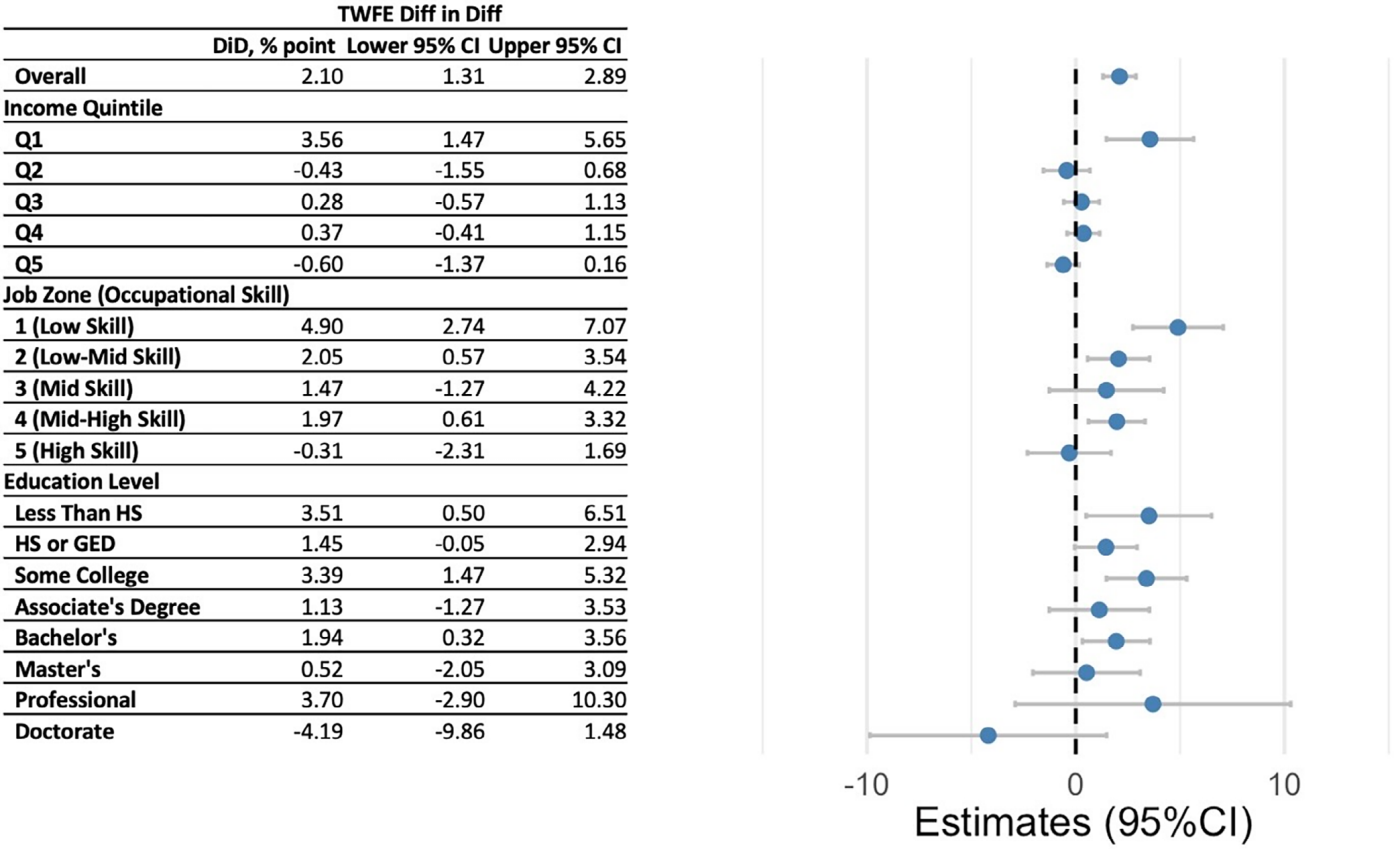
Percent difference in difference in net income between expansion and nonexpansion states. Percent difference in differences in net income after credit deductions for each income quintile, occupation skill, and education level are shown. Net income was calculated by subtracting federal tax liability after tax credit deductions from total (pretax) income. All models were estimated using the method of de Chaisemartin and D'Haultfoeuille [36, 37, 38] and were adjusted for respondent‐reported sex (male, female), race/ethnicity (white, black, Hispanic, Asian, other), nativity (U.S. born, foreign born), and marital status (single, married and spouse present, married and spouse absent, separated, divorced, widowed). Occupational skill and educational attainment were controlled for when these factors were not the main SES measure in the analysis. The 95% CIs are based on standard errors.

Figure 3 shows findings from DiD models exploring the effects of Medicaid expansion on net income, both overall and across SES. Estimates suggest a 2.10% (95% CI, 1.31–2.89) increase in net income among respondents residing in expansion versus non‐expansion states. This corresponded with a 2.68% (95% CI, 1.92–3.43) increase in total income in expansion versus nonexpansion states (Figure S1). Across income quintiles, estimates show that the lowest income quintile of respondents experienced the greatest increase in net income as a result of expansion, experiencing an increase of 3.56% (95% CI 1.47–5.65). We identified no statistically significant income change for those in the other quintiles.

By occupational skill, estimates show that net incomes increased more substantially in expansion states relative to non‐expansion states among those in low (4.90%, 95% CI 2.74–7.07), low‐mid (2.05%, 95% CI 0.57–3.54), and mid‐high (1.97%, 95% CI 0.61–3.32) skill occupations. Individuals in mid‐skill occupations (1.47%, 95% CI –1.27–4.22) saw an insignificant increase in net income, whereas high skill occupations (−0.31%, 95% CI –2.31–1.69) saw an insignificant decrease. By educational attainment, net income growth was experienced by those with less than a high school degree (3.51%, 95% CI 0.50–6.51), some college (3.39%, 95% CI 1.47–5.32), and those with a Bachelor's degree (1.94%, 95% CI 0.32–3.56). Those with a high school degree or GED (1.45%, 95% CI –0.05–2.94), Associate's (1.13%, 95% CI –1.27–3.53), master's (0.52%, 95% CI –2.05–3.09), and professional degrees (3.70%, 95% CI –2.90–10.30) saw a nonsignificant income increase as a result of expansion, whereas doctorate degree holders (−4.19%, 95% CI –9.86–1.48) saw a nonsignificant decrease.

**FIGURE 4:**
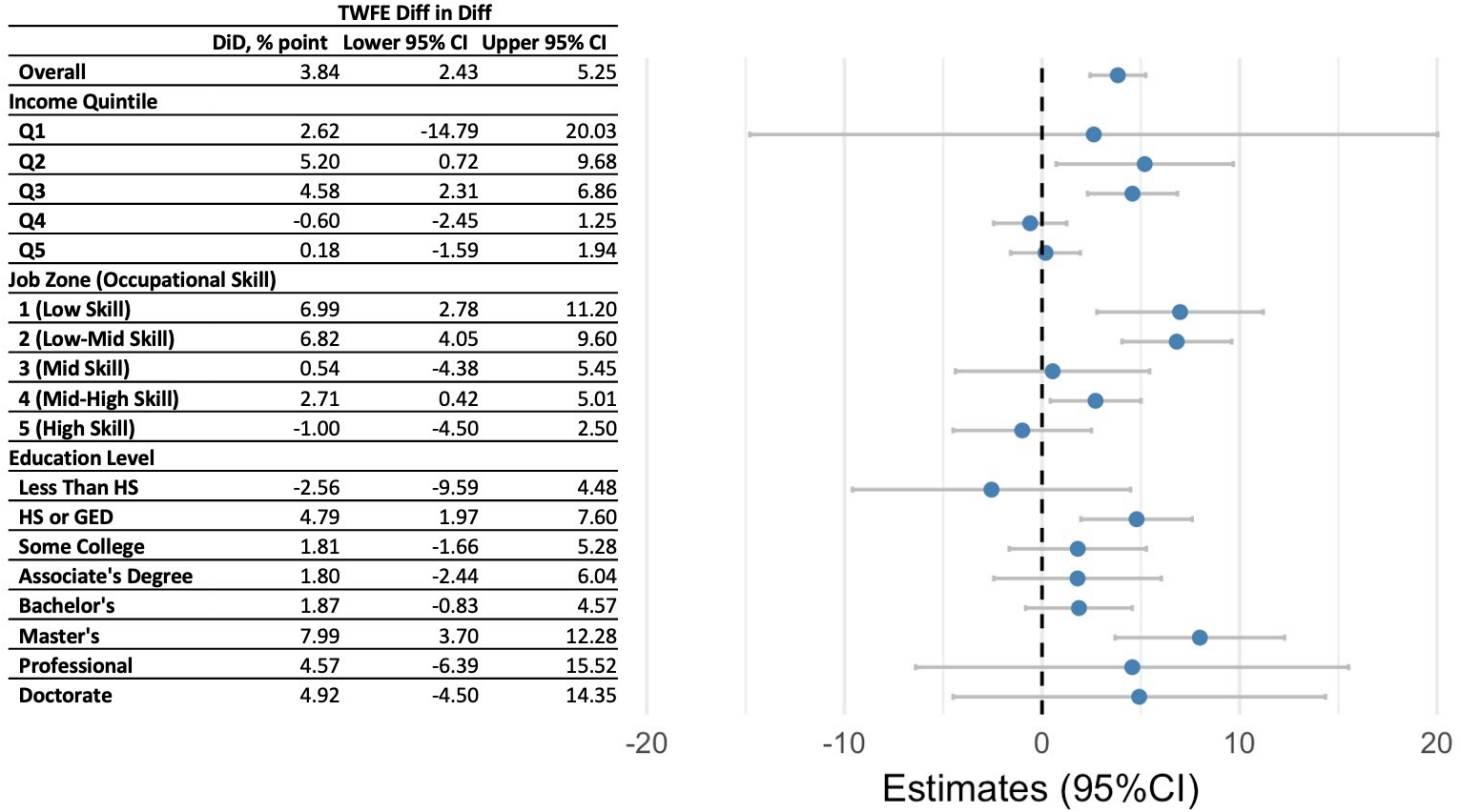
Percent difference in difference in federal tax liability between expansion and nonexpansion states. Percent difference in differences in federal tax liability after credit deductions for each income quintile, occupation skill, and education level are shown. All models were estimated using the method of de Chaisemartin and D′Haultfoeuille [36, 37, 38] and were adjusted for respondent‐reported sex (male, female), race/ethnicity (white, black, Hispanic, Asian, other), nativity (U.S. born, foreign born), and marital status (single, married and spouse present, married and spouse absent, separated, divorced, widowed). Occupational skill and educational attainment were controlled for when these factors were not the main SES measure in the analysis. The 95% CIs are based on standard errors.

Figure 4 presents analysis of federal tax liability change. Overall, we found a statistically significant change in federal tax liability (3.84%, 95% CI 2.43–5.25) as a result of expansion. Those in the lowest income quintile experienced an insignificant 2.62% (95% CI –14.79–20.03) increase in federal tax liability. The wide confidence interval likely reflects the presence of all those with a zero or negative tax liability in this quintile. The second (5.20%, 95% CI 0.72–9.68) and third (4.58%, 95% CI 2.31–6.86) quintiles experienced significant increases in federal tax liability. The changes in the fourth (−0.60%, 95% CI –2.45–1.25) and fifth (0.18%, 95% CI –1.59–1.94) income quintiles were insignificant. Low occupational skill workers saw a 6.99% (95% CI 2.78–11.20) increase, low‐mid skill workers saw a 6.82% (95% CI 4.05–9.60) increase, and mid‐high skill workers saw a 2.71% (95% CI 0.42–5.01) increase in federal tax liability. Mid skill (0.54%, 95% CI –4.38–5.45) and high skill (−1.00%, 95% CI –4.50–2.50) workers saw insignificant changes in their tax liability. By educational groups, those with a high school degree or GED (4.79%, 95% CI 1.97–7.60) and those with a master's (7.99%, 95% CI 3.70–12.28) saw significant increases in tax liability. Tax liability did not change significantly as a result of expansion for any other education levels.

## 4 Discussion

Event study findings indicate an overall trend of net income gains with increased federal tax liability in Medicaid expansion states over time. Still, findings from DiD models suggest that compared to individuals in nonexpansion states, those residing in expansion states experienced income gains as a result of expansion, even after federal tax liability was taken into consideration. Furthermore, both income and tax liability gains appear to have been disproportionately experienced by certain SES groups. Namely, some of the greatest increases in income and federal tax liability were realized by the lowest income quintiles of respondents, by those with lower levels of education, and by lower occupational skill workers. As individuals with lower levels of education and those employed in lower skill occupations also had lower incomes on average, these SES groups likely benefited the most because they were most likely to be newly eligible for Medicaid following the Affordable Care Act expansion. Our results therefore suggest that Medicaid expansion might benefit newly eligible individuals not only through increasing their access to healthcare, but also through improving their earning capacity, which may have positive downstream effects for their health in turn. Potential mechanisms linking Medicaid expansion to higher incomes for lower SES individuals could include bolstered health outcomes that enable individuals to work more hours or obtain qualitatively better occupations. Indeed, obtaining Medicaid insurance might offer greater flexibility in seeking better job opportunities without fear of losing health coverage [22].

Although by smaller margins, findings suggest several SES groups also had significant increases in net income, including the mid‐high occupation skill workers and those with either “some college” or Bachelor's degrees. Any findings showing any SES groups having reduced net income after expansion were insignificant. This finding in particular suggests that the highest earning, most highly educated individuals, alongside those working in the highest skill occupations, are not overwhelmingly subsidizing safety net programs, and a large portion of those who are highly skilled or educated benefit economically or are at the very least unimpeded by Medicaid expansion.

Regarding our analysis of tax liabilities, we found that the second and third quintiles of earners experienced the only significant increase in liability as a result of expansion, while no income quintile group saw a significant decrease in liability. Those in the highest income quintile also experienced increased tax liability following expansion; however, confidence intervals for these estimates were very large. This finding could be due to the fact that most zero and negative federal tax liabilities were reported in this quintile, making the mean federal tax liability so small that the same differences in tax liability are proportionally much greater. At the same time that tax liabilities increased for lower earners, those in the fourth income quintile experienced an insignificant tax liability reduction. The fifth quintile of earners also reported increased tax liabilities, but this finding was insignificant and reflected the smallest absolute change in tax liability among all SES groups.

Our findings that the upper and middle quintiles of earners did not owe more in federal taxes as a result of expansion contradict commonly made arguments from policymakers and high‐income individuals that increased governmental spending on healthcare and other safety net programs necessitates a significant uptick in tax liability for high‐SES groups. On the contrary, our findings suggest that the taxes used to fund Medicaid expansion are not disproportionately drawn from high‐SES groups, but rather from lower‐SES individuals, who are also most likely to benefit from expansion. Furthermore, the statistically significant increase in federal tax liabilities among all earners suggests that increased spending on Medicaid expansion resulted in economic stimulation, particularly among lower SES groups, perhaps through increasing their capacity to contribute to the federal tax base.

## 5 Limitations

Several limitations of this study warrant future scholarly attention. First, we investigated the effects of Medicaid expansions that occurred under the ACA, not during expansions that occurred prior to ACA implementation. Consequently, our findings are not generalizable to the period prior to ACA implementation. Second, although the quasi‐experimental design employed in this study offers several methodological strengths, unobserved state‐year trends may still confound the relationship between expansion and the economic outcomes investigated if those trends are systematically related to both the likelihood of Medicaid expansion and changes in income or tax liability. Third, our analyses are unable to capture state or local tax liabilities, as this information is not collected by the CPS. Fourth, some research suggests that survey‐based estimates of income, such as those available in the CPS, may be systematically biased, with respondents underreporting their incomes. This issue has been shown to be particularly problematic among low‐income populations [49]. As a result, the effects of ACA Medicaid expansions on income identified in our analysis—particularly among low SES groups—may be overestimated. Future work may consider using administratively linked survey data to capture more precise estimates of income and other economic outcomes.

Finally, although we propose several potential mechanisms driving the relationship between ACA Medicaid expansions and income/tax liability, testing the specific pathways through which this relationship operates was outside of the scope of this analysis. Future research aimed at demonstrating a causal link between Medicaid expansion and changing economic outcomes should further investigate the potential factors that might mediate this relationship.

## 6 Conclusions

Overall, our analyses suggest that ACA Medicaid expansions provided overall economic benefits to states' populations in the form of income gains and the creation of a larger taxation base, with the greatest increases in both income and tax liability observed among those in the lowest SES groups. Additionally, our results suggest that higher income earners and those in higher‐skilled and educated positions did not experience income losses or increased tax liability as a result of these expansions, as *all* SES groups experienced inflation‐adjusted income gains, and many of the wealthiest groups had decreases in federal tax liability.

Findings also further understanding of the role that Medicaid expansion plays in shaping economic and health inequality more broadly. Continued expansions to government‐funded healthcare might confer even greater health and economic benefits to the U.S. population [50]. Increasing earnings among those with lower SES while maintaining high earnings and stable levels of taxation among high SES groups can work to reduce existing earnings gaps and income inequality. Further expansion of government healthcare could also boost worker productivity by promoting healthy lifespan throughout the life course, which would provide support to an aging population [51]. Finally, further expansions may also dramatically reduce healthcare costs by endowing individuals with the economic resources to access and utilize healthcare for the purpose of preventive care. Such economic benefits could dramatically reduce financial burdens on hospitals [31, 34] while also mitigating health disparities across socioeconomic groups and state/local contexts [29, 32].

Overall, our findings contribute to a growing body of evidence that suggests expansions to social safety net programs (e.g., minimum wage raises, expansions to the Earned Income Tax Credit program) have important “spillover effects” that benefit the overall population and extend beyond those eligible for a given program [25, 26, 27, 28]. Our results surrounding the effects of expansion on net federal tax liability and net income across SES provide a meaningful blueprint for investigating the effects of other social safety net policies across SES groups. Future research should continue to explore whether and to what degree health‐related policies shape various aspects of population well‐being, with a particular focus on the effects of these policies across SES.

## Funding

This work was supported by the National Institutes of Health (T32GM152319).

## Conflicts of Interest

The authors declare no conflicts of interest.

## Supporting information


**Table S1:** Income, unadjusted differences.
**Table S2:** Federal tax liability, unadjusted differences.
**Table S3:** Net income, unadjusted differences.
**Figure S1:** Percent difference in difference in total income between expansion and nonexpansion states.
**Figure S2:** Event study analysis of change in total personal income pre‐ and post‐Medicaid expansion.
**Figure S3:** Event study analysis stratified by income quintile.
**Figure S4:** Event study analysis stratified by occupational skill level.
**Figure S5:** Event study analysis stratified by education level.

## Data Availability

The data that support the findings of this study were derived from the following resources available in the public domain: IPUMS CPS, https://cps.ipums.org/cps/index.shtml.
