# Supplementary material for: Who Pays, Who Gains? ACA Medicaid Expansions and Changes in Net Income and Tax Liability by Socioeconomic Status
Source: Health Serv Res. 2026 Jul 8;61(4):e70147. doi: 10.1111/1475-6773.70147 (PMC13344444; doi:10.1111/1475-6773.70147)
Supplement: Supplementary file 1 — Table S1: Income, unadjusted differences. Table S2: Federal tax liability, unadjusted differences. Table S3: Net income, unadjusted differences. Figure S1: Percent difference in difference in total income between expansion and nonexpansion states. Figure S2: Event study analysis of change in total personal income pre‐ and post‐Medicaid expansion. Figure S3: Event study analysis stratified by income quintile. Figure S4: Event study analysis stratified by occupational skill level. Figure S5: Event study analysis stratified by education level. [file HESR-61-e70147-s001.docx]

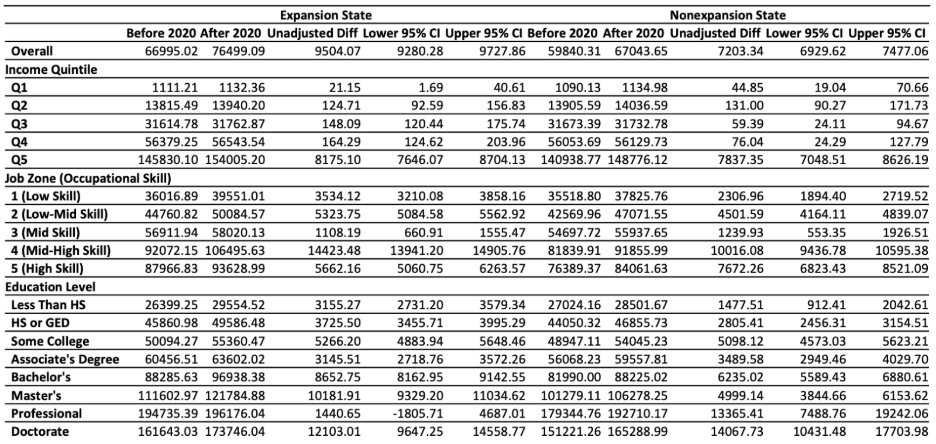
Supplemental Table 1: Income, Unadjusted Differences

Note. Unadjusted differences were calculated by subtracting the mean income after 2020 from the mean income before 2020, grouped by expansion status and stratified by SES group. 95% confidence intervals were calculated using standard errors.


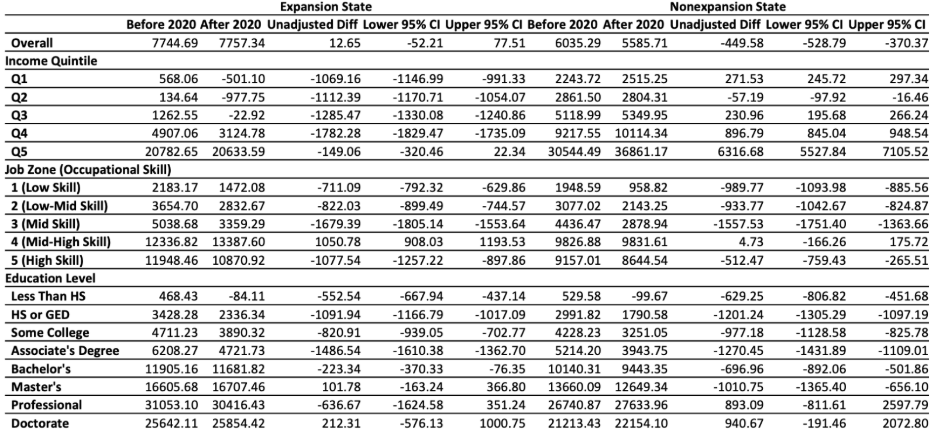


Supplemental Table 2: Federal Tax Liability, Unadjusted Differences

Note. Unadjusted differences were calculated by subtracting the mean federal tax liability after credit deductions after 2020 from the mean federal tax liability after credit deductions before 2020, grouped by expansion status and stratified by income quintile, occupation skill, and education level. 95% confidence intervals were calculated using standard errors.


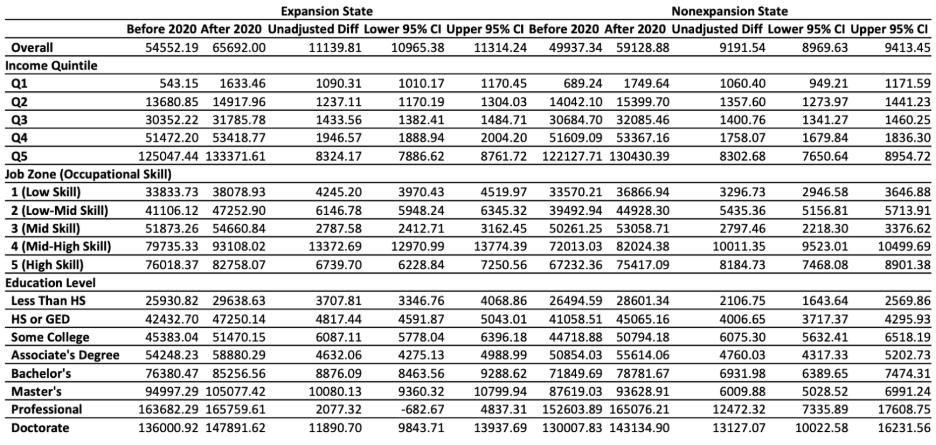


Supplemental Table 3: Net Income, Unadjusted Differences

Note. Take home income was calculated by subtracting federal tax liability after credit deductions from total personal income. Unadjusted differences were calculated by subtracting the mean net income after 2020 from the mean net income before 2020, grouped by expansion status and stratified by income quintile, occupation skill, and education level. 95% confidence intervals were calculated using standard errors.


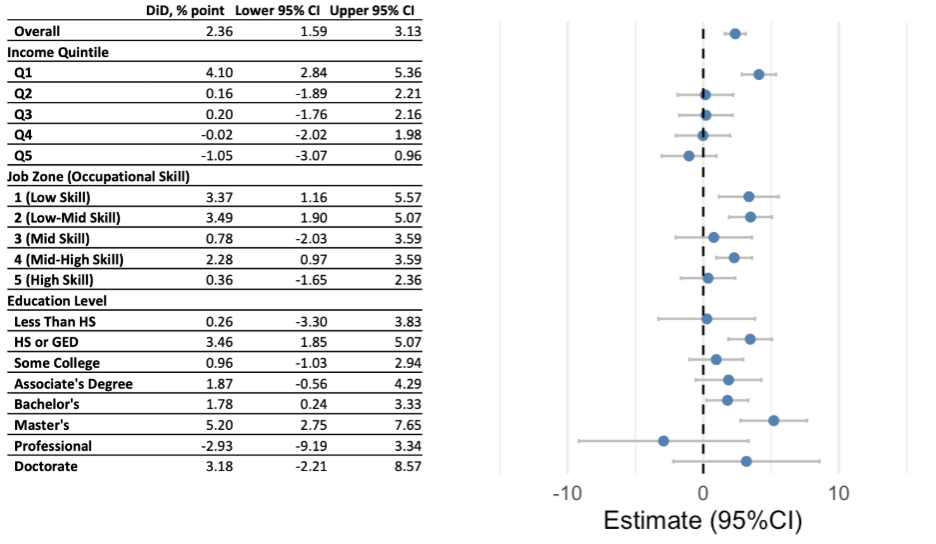
Supplemental Figure 1. Percent Difference in Difference in Total Income between Expansion and Non-expansion States

Note. Percent difference in differences in income for each income quintile, occupation skill, and education level are shown. Estimates are scaled to reflect changes in percent for income. All models were estimated using the method of de Chaisemartin and D’Haultfoeuille^36-38^ and were adjusted for respondent age, educational attainment, sex, and race and ethnicity; time invariant state-level fixed effects; and national calendar year trends. including respondent-reported sex (male, female), race/ ethnicity (white, black, Hispanic, Asian, other), nativity (U.S. born, foreign born), and marital status (single, married and spouse present, married and spouse absent, separated, divorced, widowed). Occupational skill and educational attainment were controlled for when these factors were not the main SES measure in the analysis. The 95% CIs are based on standard errors.


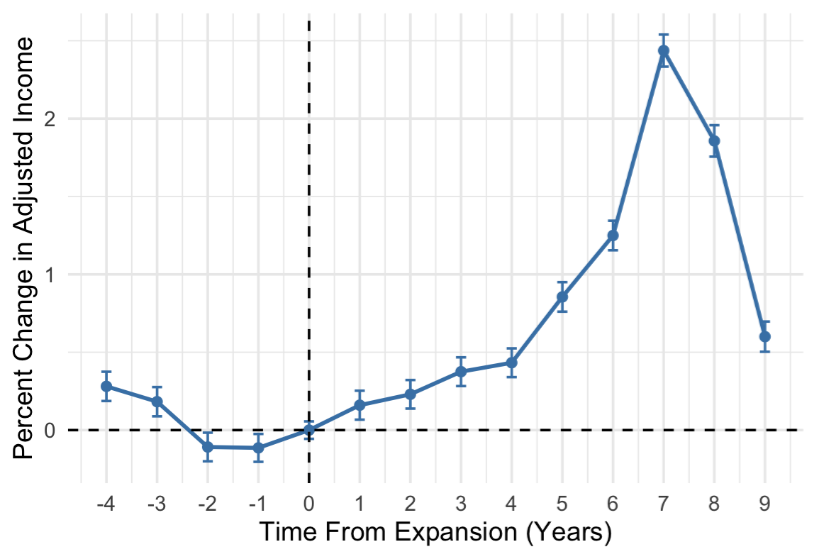


Supplemental Figure 2: Event Study Analyses of Change in Total Personal Income Pre- and Post-Medicaid Expansion

Note. This figure reports an event study analysis showing percent change in total income relative to expansion year for all states that expanded Medicaid. The y-axis plots percent difference in income or federal tax liability from the year of expansion for each event time. The x-axis represents the number of years relative to the timing of state Medicaid expansions. The 95% CIs are based on standard errors. The vertical black dotted line represents the year of Medicaid expansion. The horizonal black dotted line represents the income or federal tax liability the year of Medicaid expansion.


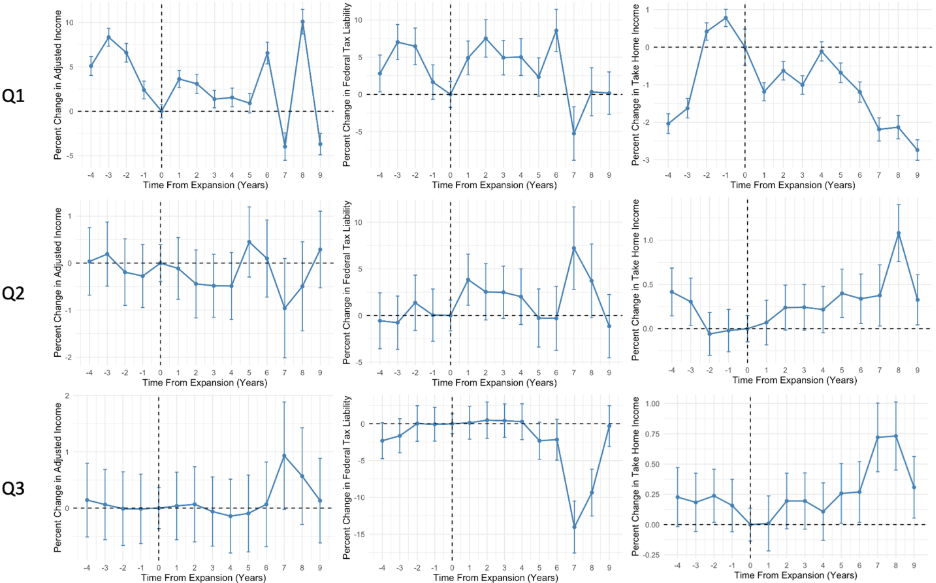

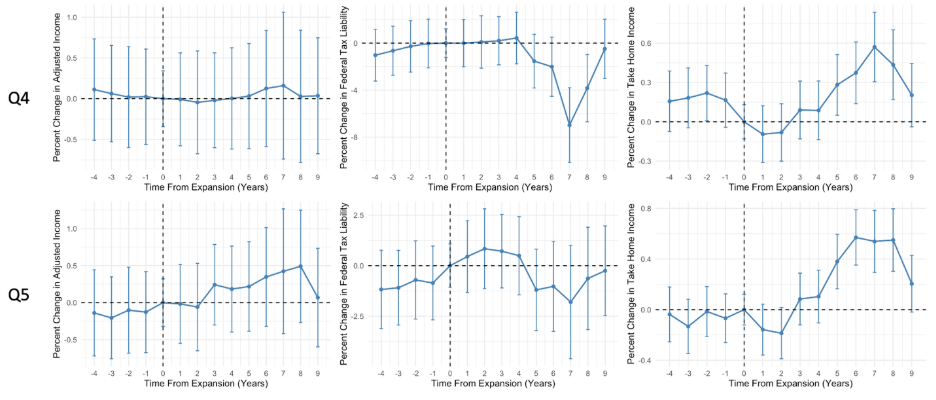


Supplemental Figure 3: Event Study Analysis Stratified by Income Quintile

Note. Event study analyses show percent change in income relative to expansion year for all states that expanded Medicaid by income quintile. The y-axis plots percent difference in income, federal tax liability after credit deductions, or net income from the year of expansion for each event time. The x-axis represents the number of years relative to the timing of state Medicaid expansions. The 95% CIs are based on standard errors. The vertical black dotted line represents the year of Medicaid expansion. The horizonal black dotted line represents the income, federal tax liability, or net income the year of Medicaid expansion.


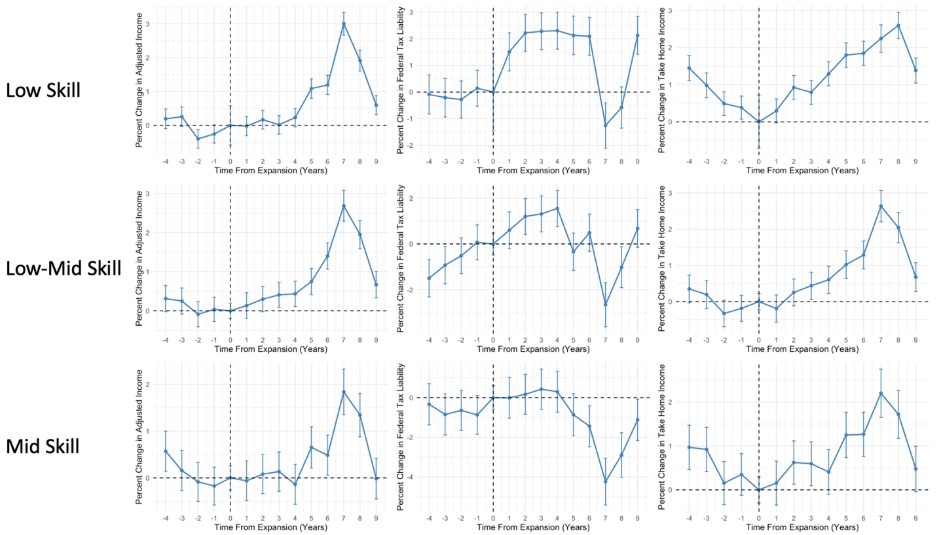

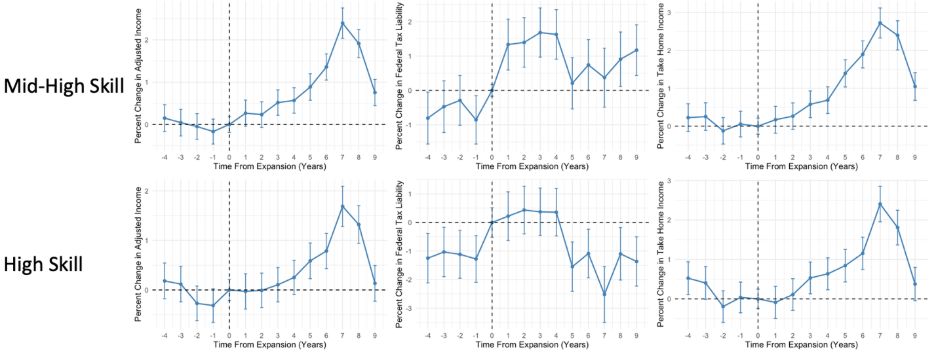


Supplemental Figure 4: Event Study Analysis Stratified by Occupational Skill Level

Note. Event study analyses show percent change in income relative to expansion year for all states that expanded Medicaid by occupational skill level. The y-axis plots percent difference in income, federal tax liability after credit deductions, or net income from the year of expansion for each event time. The x-axis represents the number of years relative to the timing of state Medicaid expansions. The 95% CIs are based on standard errors. The vertical black dotted line represents the year of Medicaid expansion. The horizonal black dotted line represents the income, federal tax liability, or net income the year of Medicaid expansion.


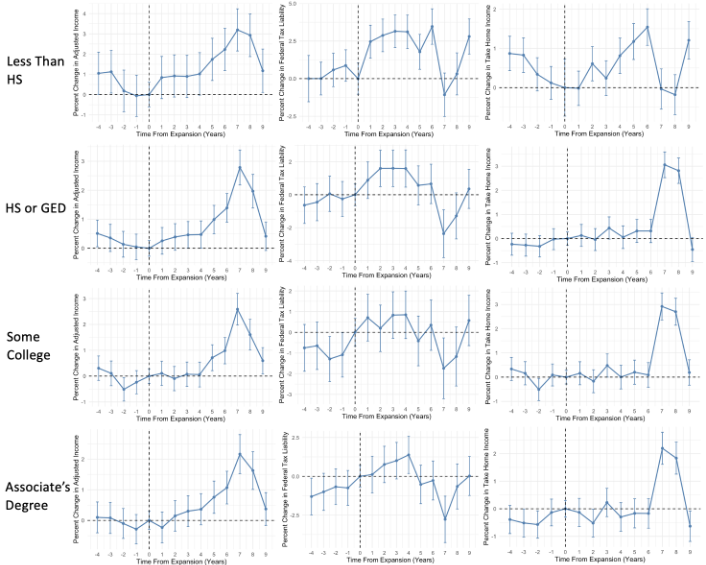

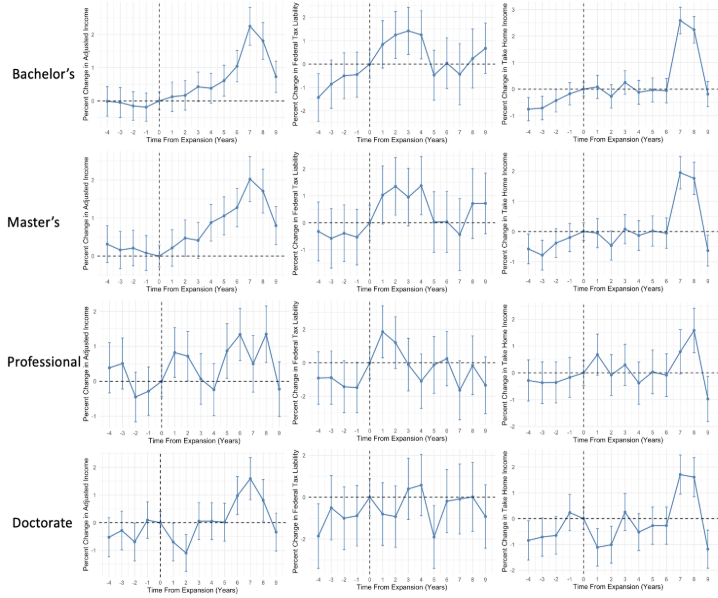


Supplemental Figure 5: Event Study Analysis Stratified by Education Level

Note. Event study analyses show percent change in income relative to expansion year for all states that expanded Medicaid by education level. The y-axis plots percent difference in income, federal tax liability after credit deductions, or net income from the year of expansion for each event time. The x-axis represents the number of years relative to the timing of state Medicaid expansions. The 95% CIs are based on standard errors. The vertical black dotted line represents the year of Medicaid expansion. The horizonal black dotted line represents the income, federal tax liability, or net income the year of Medicaid expansion.
